# Comparative plastid genomics reveals phylogenetic relationships and candidate molecular markers in *Salvia*

**DOI:** 10.3389/fpls.2026.1932456

**Published:** 2026-09-14

**Authors:** Chengwen Gao, Yujie He, Mingxuan Wu

**Affiliations:** Medical Research Center, The Affiliated Hospital of Qingdao University, Qingdao University, Qingdao, Shandong, China

**Keywords:** molecular markers, phylogenomics, plastid genome, RNA editing, *Salvia*

## Abstract

**Introduction:**

Salvia is the largest genus in Lamiaceae and includes numerous medicinal, ornamental, and aromatic species. Species identification and rational resource utilization in this genus remain difficult because morphological characters are variable, infrageneric boundaries are sometimes ambiguous, and broad comparative genomic resources are still limited. In addition, plastid RNA editing has rarely been characterized in Salvia.

**Methods:**

We assembled six new plastid genomes, compared complete plastid genomes representing major lineages of Salvia, and characterized plastid RNA editing in *Salvia officinalis*.

**Results:**

The Salvia plastid genomes were highly conserved in organization and ranged from 150,604 to 151,994 bp, with 132–134 annotated gene features and GC contents of 37.95%–38.11%. Codon usage showed a clear A/U-ending preference, with AGA being the most strongly preferred codon. A total of 623 SSRs were detected across these plastomes, with 24–45 loci per species; most were A/T-rich mononucleotide repeats and were concentrated in the LSC and intergenic regions. REPuter identified 30–50 nontandem repeats per plastome, and forward and palindromic repeats occurred at comparable frequencies. These compositional features are consistent with previous reports for Salvia and provide the descriptive baseline for the analyses that follow. Comparative analyses indicated that IR regions were more conserved than single-copy regions, whereas several highly variable loci were promising candidates for marker development; the most divergent region was not the most discriminating, and ycf1 separated 99.8% of the 4,753 Salvia species pairs against 97.6% for rbcL. Phylogenomic reconstruction based on 98 Salvia plastomes, performed after removing one inverted-repeat copy from every genome and trimming ambiguously aligned columns, recovered three major clades broadly consistent with current subgeneric classification, and the same three clades were recovered from a partitioned supermatrix of 50 protein-coding genes. In *S. officinalis*, 21 plastid RNA editing sites were identified in 10 protein-coding genes; 20 sites were nonsynonymous, and the second codon position was most frequently edited (17 of 21 sites). Seven nonsynonymous sites were confirmed by Sanger sequencing of genomic DNA and cDNA amplicons, all reported on the coding strand.

**Discussion:**

The plastid genomes of Salvia are structurally conservative but contain sufficient localized variation for marker development and species discrimination. The expanded plastome phylogeny improves resolution of major evolutionary lineages within the genus, and the RNA editing analysis adds a functional layer to plastid genome evolution in Salvia. Together, these results provide practical genomic resources for taxonomy, germplasm authentication, phylogenetic inference, and the sustainable utilization of Salvia resources.

## Introduction

The genus *Salvia* L., commonly recognized as sage, constitutes the largest, most diverse, and arguably the most economically significant assemblage within the mint family, Lamiaceae. Classified within the subfamily Nepetoideae and the tribe Mentheae, the genus comprises approximately 1,000 recognized species (Drew et al., 2017; Sharifi-Rad et al., 2018; Afzal et al., 2025). Members of the genus are of substantial medicinal, horticultural, ecological, and commercial value. Many species are used in traditional medicine, as sources of essential oils and bioactive metabolites, or as ornamental crops (Afzal et al., 2025; Nicolescu et al., 2026). This broad utility has made accurate species identification and rational resource evaluation increasingly important.

Despite its importance, *Salvia* remains taxonomically challenging. Morphological characters can be plastic, closely related species may be difficult to distinguish, and some infrageneric boundaries remain unstable or incompletely resolved (Will and Classen-Bockhoff, 2017; Rose et al., 2021). These problems directly affect medicinal authentication, conservation planning, and the efficient exploitation of germplasm resources. Broader genomic comparisons are therefore needed to strengthen species delimitation and evolutionary inference across the genus.

Plastid genomes provide an effective system for such analyses because they are typically conserved in structure, mostly uniparentally inherited, and rich in phylogenetically informative variation at both the sequence and structural levels (Wicke et al., 2011; Wu et al., 2024). Comparative plastid genomics has already improved molecular identification, hotspot discovery, and phylogenetic reconstruction in *Salvia* and other taxonomically complex plant groups (Wu et al., 2021; Akrami et al., 2025; Du et al., 2025). Our own recent survey of 18 *Salvia* plastomes established a comparative framework for the genus and reported a first set of plastid RNA editing sites (Gao et al., 2026); the present study builds directly on that work by adding six previously unsequenced plastomes, by extending the phylogenomic sampling to 98 *Salvia* taxa, and by re-examining plastid RNA editing with a strand-aware analysis and matched genomic controls. However, comparisons spanning multiple major *Salvia* lineages are still limited, and a broader framework is needed to connect plastome variation with species identification and resource utilization.

In parallel with sequence variation, RNA editing adds a post-transcriptional layer of functional diversity to plant organelle genomes. In plastids, editing is dominated by C-to-U conversions and can alter codons, restore conserved amino acids, and modulate transcript and protein function (Ichinose and Sugita, 2017; Duan et al., 2023; Knoop, 2023). Although plastid RNA editing has been studied in several angiosperms, its landscape in *Salvia* remains poorly characterized.

In this study, we newly assembled six complete plastid genomes and combined them with 12 previously available plastomes representing major *Salvia* lineages for comparative analyses. In addition, we investigated plastid RNA editing in *S. officinalis*. Specifically, we aimed to: (1) characterize genome structure, gene content, codon usage, and repetitive sequences in 18 *Salvia* plastomes; (2) identify highly variable regions suitable for molecular marker development and quantify how well they discriminate *Salvia* species relative to the standard plastid barcodes; (3) resolve phylogenetic relationships using an expanded plastome dataset in which inverted repeat redundancy and ambiguously aligned regions are removed before inference; and (4) depict the plastid RNA editing profile of *S. officinalis* on the coding strand of each edited gene. These results provide updated genomic resources for species identification, phylogenetic studies, and the conservation and utilization of *Salvia* germplasm.

## Materials and methods

### Plant materials, sequencing data, and plastome assembly

The six newly assembled *Salvia* plastomes were reconstructed from publicly available target-enrichment (anchored hybrid enrichment) sequencing datasets generated for phylogenetic studies of *Salvia* (BioProjects PRJNA773953 and PRJNA1113632; Kriebel et al., 2019; Rose et al., 2021), in which plastid reads occur as abundant off-target sequence. The paired-end libraries were sequenced on the Illumina HiSeq 2500 platform with a read length of 2 x 150 bp. No new plant material was collected for the plastome assemblies; every source sample is a vouchered specimen or living accession, and the voucher or isolate identifier, tissue, developmental stage, BioSample, BioProject and run accession of each dataset are listed in **Supplementary Table 1**. Leaf tissue of one cultivated *Salvia officinalis* plant was collected for the Sanger validation of the RNA editing sites, as described below. Twelve published plastomes were added for comparison, chosen so that all currently recognized subgenera of *Salvia* sensu lato, both New World and Old World lineages, and the segregate genera now placed within *Salvia* (*Audibertia*, *Dorystaechas*, *Perovskia* and *Rosmarinus*) were represented; the subgeneric coverage of this 18-plastome comparative set is given in **Supplementary Table 9**. The accession and source of every plastome are given in **Table 1**.

**Table 1:** Plastid genomic characteristics of the 18 *Salvia* species compared in this study.

Species	Genome (bp)	LSC (bp)	IR (bp)	SSC (bp)	Total annotated gene features	Number of CDS	Number of tRNA genes	Number of rRNA genes	GC content (%)	Source	Accession
*Salvia brandegeei*	151,340	82,524	25,605	17,606	132	87	37	8	38.11	This study	PX971244
*Salvia clevelandii*	151,384	82,570	25,600	17,614	132	87	37	8	38.09	This study	PX971245
*Salvia columbariae*	151,338	82,586	25,611	17,530	132	87	37	8	38.10	This study	PX971246
*Salvia munzii*	151,311	82,481	25,610	17,610	132	87	37	8	38.11	This study	PX971247
*Salvia omeiana*	151,218	82,398	25,596	17,628	132	87	37	8	38.01	This study	PX971248
*Salvia tuerckheimii*	151,058	82,188	25,668	17,534	132	87	37	8	37.99	This study	PX971249
*Salvia apiana*	151,550	82,983	25,539	17,489	133	87	37	8	38.06	GenBank	NC_084049
*Salvia chinensis*	151,458	82,768	25,557	17,576	132	87	37	8	38.02	GenBank	PV591959
*Salvia dorystaechas*	151,994	83,112	25,619	17,644	134	87	37	8	38.03	GenBank	NC_084050
*Salvia miltiorrhiza*	151,328	82,695	25,539	17,555	133	87	37	8	38.02	GenBank	NC_020431
*Salvia nilotica*	151,450	82,740	25,623	17,464	134	88	37	8	37.99	GenBank	NC_050898
*Salvia officinalis*	151,135	82,463	25,586	17,500	133	87	37	8	38.04	GenBank	MN520021
*Salvia plebeia*	151,062	82,454	25,555	17,498	133	87	37	8	38.01	GenBank	NC_050929
*Salvia rosmarinus*	151,577	82,644	25,657	17,619	132	87	37	8	38.08	GenBank	OM691672
*Salvia sclarea*	151,268	82,535	25,569	17,595	132	87	37	8	37.95	GenBank	NC_050900
*Salvia splendens*	150,604	82,181	25,283	17,857	132	87	37	8	38.04	GenBank	NC_050901
*Salvia yangii*	151,473	82,701	25,603	17,566	134	87	37	8	38.05	GenBank	MT012421
*Salvia yunnanensis*	151,413	82,656	25,590	17,577	132	87	37	8	38.02	GenBank	NC_050903

Raw reads were filtered with Trimmomatic v0.38 (Bolger et al., 2014). High-quality reads were assembled *de novo* with SPAdes v3.6.1 (Bankevich et al., 2012), and plastid contigs were ordered against the *S. miltiorrhiza* plastid genome (NC_020431) in Geneious Prime v2025.0.2 (Kearse et al., 2012). Genome annotation was performed with CPGAVAS2 (Liu et al., 2012), refined in Sequin v12.91, and visualized with OGDRAW v1.3.1 (Greiner et al., 2019). Assembly accuracy and species identity were checked against independent data where possible: *S. columbariae* is the only one of the six species for which a conspecific plastome had already been published (PP737620), and the two sequences were aligned with MAFFT and compared base by base (**Supplementary Table 11**).

### Codon usage and repeat analyses

Relative synonymous codon usage (RSCU) values were calculated using custom scripts according to the method of Sharp and Li (Sharp and Li, 1986). SSRs were identified with MISA using minimum thresholds of 10 repeat units for mononucleotides, 5 for dinucleotides, 4 for trinucleotides, and 3 for tetra-, penta-, and hexanucleotides (Beier et al., 2017). These thresholds were selected because they are widely used and relatively conservative in plastome SSR surveys, which helps reduce over-detection of very short low-complexity repeats and ensures direct comparability among the analyzed *Salvia* chloroplast genomes. Although no *Salvia*-specific benchmark is currently available for optimizing plastid SSR cutoffs, the use of a uniform parameter set provides a consistent basis for comparative analysis; therefore, the reported SSRs should be regarded as a conservative dataset for subsequent marker screening. Non-tandem repeats were detected with REPuter using a minimum repeat size of 30 bp, a Hamming distance of 3, and forward and palindromic repeat types (Kurtz et al., 2001). A Hamming distance of 3 was used to permit limited mismatches in the identification of imperfect repeats, thereby improving sensitivity to biologically relevant repeat variation while avoiding overly permissive matches. Together with the 30-bp minimum repeat size, this setting provided a balanced and relatively conservative criterion for repeat detection and was applied uniformly across all analyzed *Salvia* chloroplast genomes to ensure comparability.

### Comparative plastome analyses

IR/SC junctions were examined with IRscope (Amiryousefi et al., 2018). Whole plastome alignments were generated with MAFFT v7.490 (Katoh and Standley, 2013). Comparative sequence conservation was visualized in mVISTA with *S. aethiopis* (PV870855) as the reference, a *Salvia* plastome deliberately chosen from outside the 18 so that no member of the comparative set serves as its own baseline (Frazer et al., 2004). These whole-genome visual comparisons use the 18-plastome set, because a larger matrix cannot be displayed legibly; the nucleotide-diversity and phylogenetic analyses below use all 98 *Salvia* plastomes. Nucleotide diversity was calculated in DnaSP v6.12 using a sliding window of 600 bp and a step size of 200 bp (Librado and Rozas, 2009). For each candidate marker region, the number of *Salvia* species pairs resolved by that region alone was counted across the 98-plastome alignment and compared with the corresponding values for *rbcL* and *matK* (**Supplementary Table 10**).

### Phylogenetic reconstruction

For phylogenetic analysis, 98 complete plastid genomes of *Salvia* were analyzed together with *Mentha longifolia* (ON124917) and *M. suaveolens* (NC_086486) as outgroups (**Supplementary Table 7**). Because retaining both copies of the inverted repeat doubles the weight of the most conserved third of the plastome, the inverted repeat was located *de novo* in each of the 100 genomes by exact inverted-repeat detection and one copy (IRa) was removed; the LSC, IR and SSC lengths recovered by this procedure reproduced the values reported in **Table 1**. The IR-deduplicated sequences were aligned with MAFFT v7.490 and ambiguously aligned columns were removed with trimAl v1.5 using the -automated1 heuristic (Capella-Gutierrez et al., 2009). Maximum likelihood analysis was conducted in IQ-TREE v3.0.1 (Nguyen et al., 2015), the best-fit substitution model was selected by ModelFinder under BIC (Kalyaanamoorthy et al., 2017), and node support was evaluated with 1,000 ultrafast bootstrap replicates (Hoang et al., 2018) and 1,000 SH-aLRT replicates (Guindon et al., 2010). Two additional matrices were analyzed under the same protocol to test the sensitivity of the result to these choices: complete plastomes without IR removal or trimming, which reproduces the conventional approach, and a partitioned supermatrix of the 50 protein-coding genes present in single copy in all 100 taxa, each aligned and trimmed separately; the two supporting topologies are shown in **Supplementary Figures 4** and **5**. Topologies were compared by Robinson-Foulds distance, and alignment and tree statistics for the three datasets are given in **Supplementary Table 15**.

### Identification of RNA editing sites using RNA-Seq data

Plastid RNA editing was characterized in *S. officinalis* using matched public RNA-seq (SRR5150710; experiment SRX2468800) and DNA-seq (SRR6940041; experiment SRX3884425) data from the same BioSample (SAMN06200326, BioProject PRJNA359989). Both libraries were prepared from leaf tissue of a vegetative-stage plant. The published *S. officinalis* plastid genome MN520021 was used as the reference throughout, and gene coordinates were taken from its GenBank annotation; every coding sequence was translated before use and any feature that did not yield a complete open reading frame was excluded. RNA-seq reads were aligned with HISAT2 v2.1.0 (Kim et al., 2019) with the maximum intron length set to 3,000 bp, because the longest cis-intron in a plastid genome is the *trnK* intron at about 2.5 kb and longer spliced gaps are alignment artefacts; a position spanned by a spliced gap carries no aligned base and was excluded from the depth counts. DNA-seq reads were aligned with Bowtie2 v2.3.5 (Langmead and Salzberg, 2012). Base counts were obtained from samtools mpileup with no depth cap and a minimum base quality of 20 (Danecek et al., 2021). Because *ndhB*, *rps7*, *rpl2*, *rpl23* and *ycf2* are duplicated in the inverted repeat and an IR-ambiguous read is placed in only one copy by the aligner, base counts at equivalent positions of the two IR copies were pooled before calling and each site is reported once. Every cytosine on the coding strand of every coding sequence was then tested, so that C-to-U events are always reported as C-to-U rather than as G-to-A for genes on the minus strand. A site was retained when the RNA depth was at least 20, at least five reads carried U, the editing frequency was at least 5%, the excess of U reads was significant against a sequencing-error rate estimated empirically from all non-cytosine coding positions after Benjamini-Hochberg correction (q < 0.01), and the matched DNA-seq library showed the position to be homozygous cytosine at a depth of at least 10, which excludes genomic polymorphism including at fully edited sites. Retained sites were used to summarize editing type, codon position, amino acid consequences and editing efficiency. Seven of the nonsynonymous sites, distributed across *matK*, *petB*, *rpoA* and *ndhB*, were validated by Sanger sequencing. Validation used an independent biological sample: fresh leaves collected from a cultivated *Salvia officinalis* plant grown in Qingdao, China. Total genomic DNA was extracted from this leaf tissue with the Ezup column plant genomic DNA purification kit (Sangon Biotech, Shanghai, China; B518261) and total RNA with a Trizol-based kit (Sangon Biotech; B511321). Six amplicons covering the seven sites were amplified in parallel from the genomic DNA with Taq Plus DNA polymerase (Sangon Biotech; B600090) and from the total RNA by one-step RT-PCR with the Evo M-MLV One Step RT-PCR Kit (Accurate Biology, Changsha, China; AG11606); the RT-PCR products are referred to as cDNA amplicons throughout. Products were gel-purified (SanPrep column kit, Sangon Biotech; B518131) and sequenced with BigDye Terminator v1.1 on an ABI 3730xl analyzer on 19 March 2026 (Sangon Biotech service orders 26SC94 for gDNA and RT2654 for cDNA). Primers, product sizes and trace identifiers are given in **Supplementary Table 8**. Both primer directions were sequenced for each cDNA amplicon and the forward primer alone for each gDNA amplicon, and the read corresponding to the coding strand of the gene is the one shown in **Figure 1**. Because this material is a different individual from the one that generated SRR5150710, the amplicons test whether the same positions are edited in *S. officinalis*, not the editing frequencies measured from the RNA-seq data. The analysis workflow is shown in **Figure 1a**.

**Figure 1:**
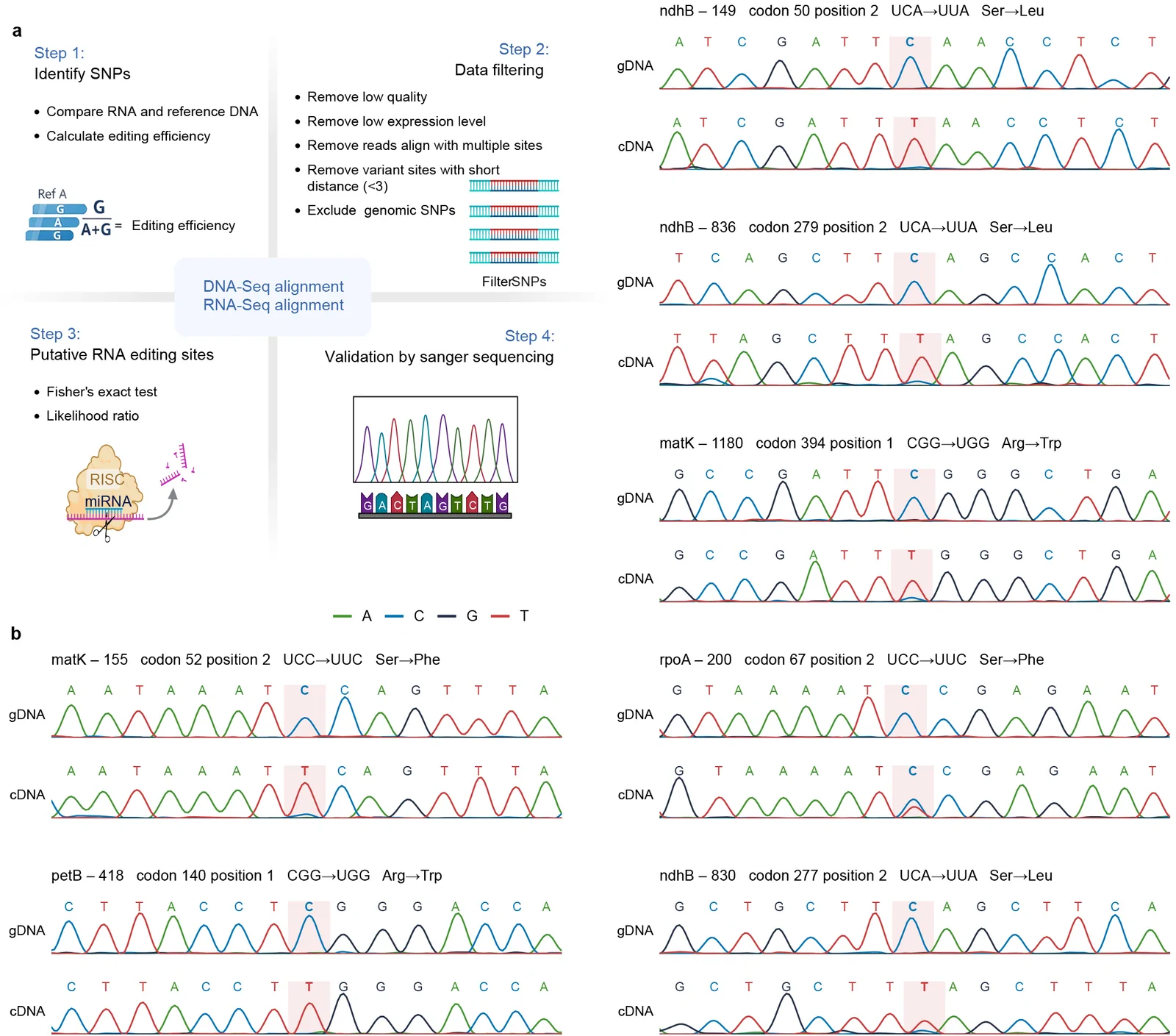
Identification and validation of plastid RNA editing sites in *Salvia officinalis* (reference plastome MN520021). **(a)** Workflow used to align the reads, enumerate the candidate cytosines on the coding strand, and filter them against the matched genomic library. **(b)** Sanger sequencing chromatograms of regions amplified from genomic DNA (gDNA) and cDNA of a single cultivated *S. officinalis* plant grown by the authors, validating seven nonsynonymous editing sites.

## Results

### Plastid genome architecture and compositional features

The 18 plastid genomes analyzed in this study comprised six newly assembled from publicly available sequencing datasets, together with twelve previously published genomes representing the major subgenera of *Salvia* retrieved from public databases; the source and accession of each is given in **Table 1**, and the subgeneric coverage of the set in **Supplementary Table 9**. All 18 plastid genomes displayed the typical quadripartite architecture consisting of an LSC region, an SSC region, and two IRs (**Figure 2**; **Supplementary Figure 1**; **Table 1**). Genome size ranged from 150,604 bp in *S. splendens* to 151,994 bp in *S. dorystaechas*. The LSC region ranged from 82,181 to 83,112 bp, the SSC region from 17,464 to 17,857 bp, and each IR from 25,283 to 25,668 bp. GC content was highly stable across species, varying only from 37.95% to 38.11%. The newly assembled *S. columbariae* plastome was 99.96% identical to the independently published conspecific plastome PP737620 across 136,977 aligned positions, compared with 99.60-99.64% identity to its closest congeners, confirming both the accuracy of the assembly and the identity of the source material (**Supplementary Table 11**).

**Figure 2:**
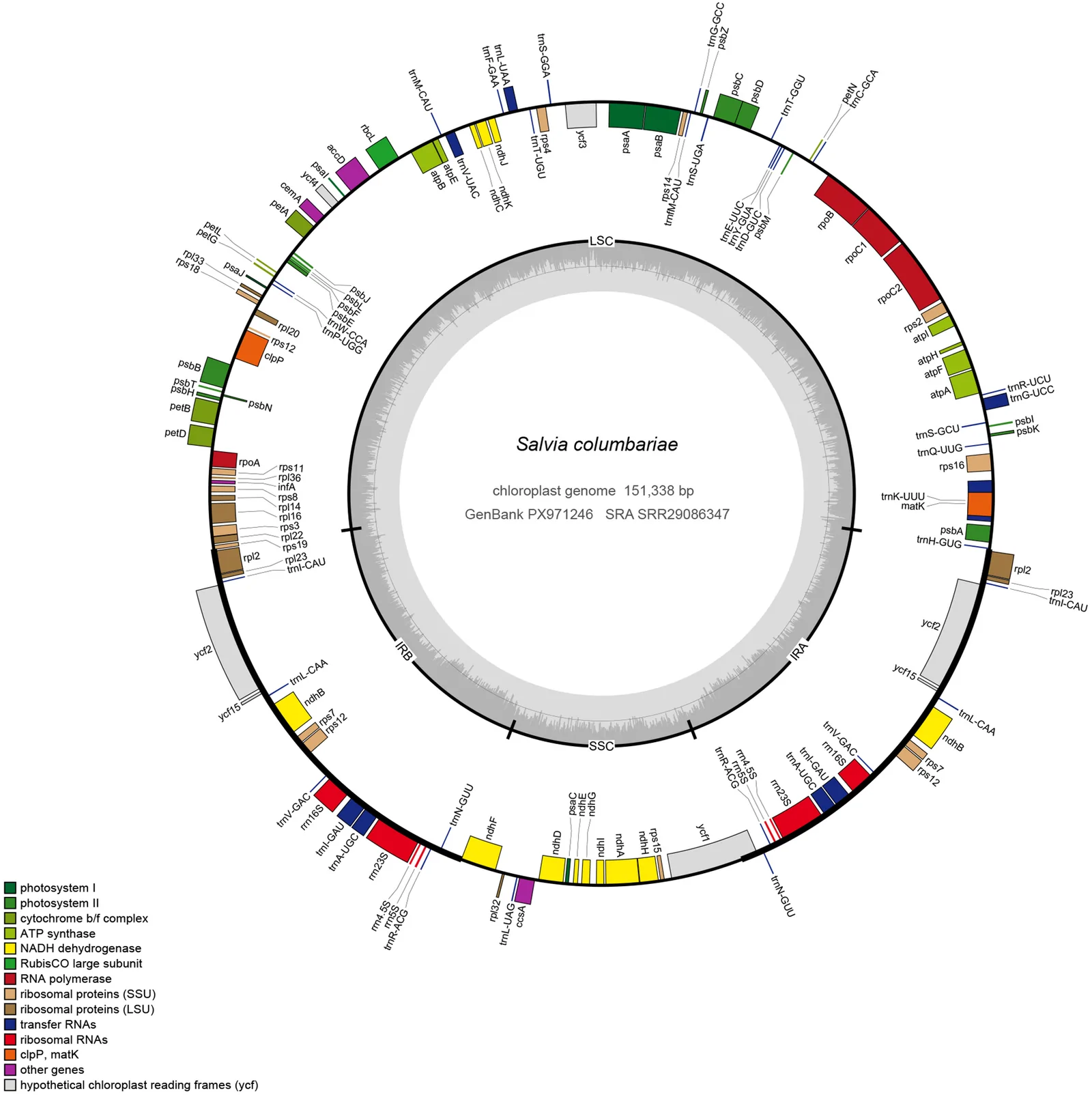
Circular map of the plastid genome of *Salvia columbariae* (GenBank PX971246), assembled in this study from SRA run SRR29086347. Genes located on the inside of the circle are transcribed in the clockwise direction, whereas those on the outside are transcribed counterclockwise. Different colors denote different functional groups.

Gene content was also conservative. The plastomes contained 132–134 annotated gene features when partial or pseudogene copies at IR boundaries were included. These comprised 87–88 CDS features, 37 tRNA genes, and 8 rRNA genes. Functional annotation grouped these genes into photosynthesis-related genes, self-replication genes, other genes, and genes of unknown function (**Supplementary Table 2**). A total of 23 genes contained introns: 19 genes harbored one intron, whereas *clpP*, *ycf3*, and the two trans-spliced copies of *rps12* contained two introns. Genes duplicated in the IRs included seven protein-coding genes, seven tRNAs, and four rRNAs. Together, these results indicate that plastid genome structure and gene complement are highly conserved across the sampled *Salvia* species.

### Codon usage bias

RSCU analysis revealed a consistent codon usage pattern across the 18 plastomes (**Figure 3**; **Supplementary Table 3**). Preferred sense codons almost exclusively ended in A or U; UUG was the only G-ending codon with mean RSCU greater than 1.0. The most strongly preferred codon was AGA (Arg), with species-specific RSCU values of 1.82-1.89. Other highly preferred codons included UUA (Leu; 1.75–1.79), GCU (Ala; 1.71–1.74), UCU (Ser; 1.67–1.71), ACU (Thr; 1.60–1.64), and UAU (Tyr; 1.59–1.61). By contrast, many GC-ending codons were underrepresented. As expected, AUG and UGG showed no codon bias (RSCU = 1.0) because they are each represented by a single codon. Codon usage in *Salvia* plastomes is therefore strongly biased toward A/U-ending synonymous codons, a pattern that matches previous reports for *Salvia* and for Lamiaceae plastomes generally and that we treat here as a confirmatory baseline rather than a new finding.

**Figure 3:**
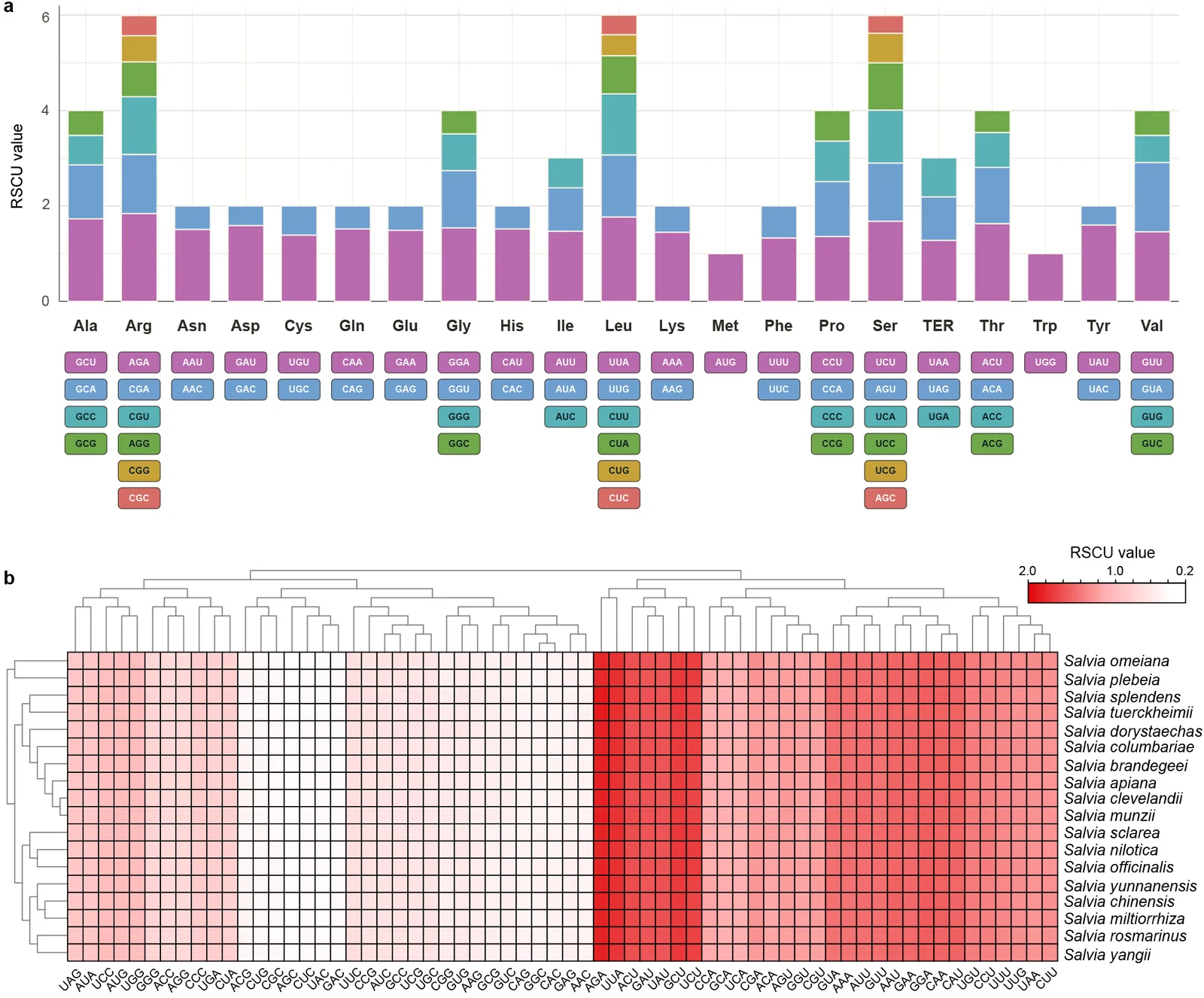
Codon usage patterns in the plastid genomes of *Salvia* species. **(a)** Codon usage bias of amino acids encoded in the plastid genomes. **(b)** Hierarchically clustered heatmap of relative synonymous codon usage (RSCU) values among *Salvia* species. Redder colors indicate stronger codon preference.

### SSRs and long repeats

SSR analysis identified 623 loci across the 18 plastomes, corresponding to 24–45 loci per species (**Supplementary Figure 2a**; **Supplementary Tables 4**, **5**). *S. omeiana* contained the highest number of SSRs (45), followed by *S. yunnanensis* (42) and *S. miltiorrhiza* (41), whereas *S. rosmarinus* had the fewest (24). Mononucleotide SSRs were the most abundant class (392/623, 62.9%), followed by tetranucleotide (120), dinucleotide (74), compound (23), trinucleotide (11), hexanucleotide (2) and pentanucleotide (1) repeats. Mononucleotide SSRs were overwhelmingly A/T-rich: 386 of 392 consisted of A or T motifs. Regionally, SSRs were concentrated in the LSC region (524 loci), followed by the SSC (89) and IR regions (10) (**Supplementary Figure 2b**). Annotation-based classification further showed that 82.0% of SSRs were located in intergenic regions, whereas 18.0% occurred within coding sequences.

REPuter detected 826 non-tandem repeats across the 18 plastomes (**Supplementary Figure 2c**; **Supplementary Table 6**). Forward repeats (407) and palindromic repeats (419) were nearly equally represented. Excluding the full-length IR matches automatically reported by REPuter, most repeat units fell in the 30–40 bp class, followed by the 41–50 bp class (**Supplementary Figure 2d**). These results indicate that *Salvia* plastomes contain modest but informative repeat variation that may be useful for marker development and structural comparison.

### Comparative plastome divergence and mutation hotspots

Comparison of IR/SC boundaries showed that the overall junction architecture was conserved across the 18 plastomes (**Supplementary Figure 3**). However, slight shifts were observed in the positions of *rps19*, *ndhF*, *ycf1*, and *trnH-GUG*, indicating small lineage-specific IR expansion and contraction events rather than major structural rearrangements. mVISTA comparison further confirmed the conservative nature of the plastomes (**Figure 4**). IR regions were more conserved than LSC and SSC regions, and non-coding sequences were generally more variable than coding regions. Several intergenic spacers showed noticeably elevated divergence, particularly *rps16*-*trnQ-UUG*, *psaA*-*ycf3*, and *rpl32*-*trnL-UAG*.

**Figure 4:**
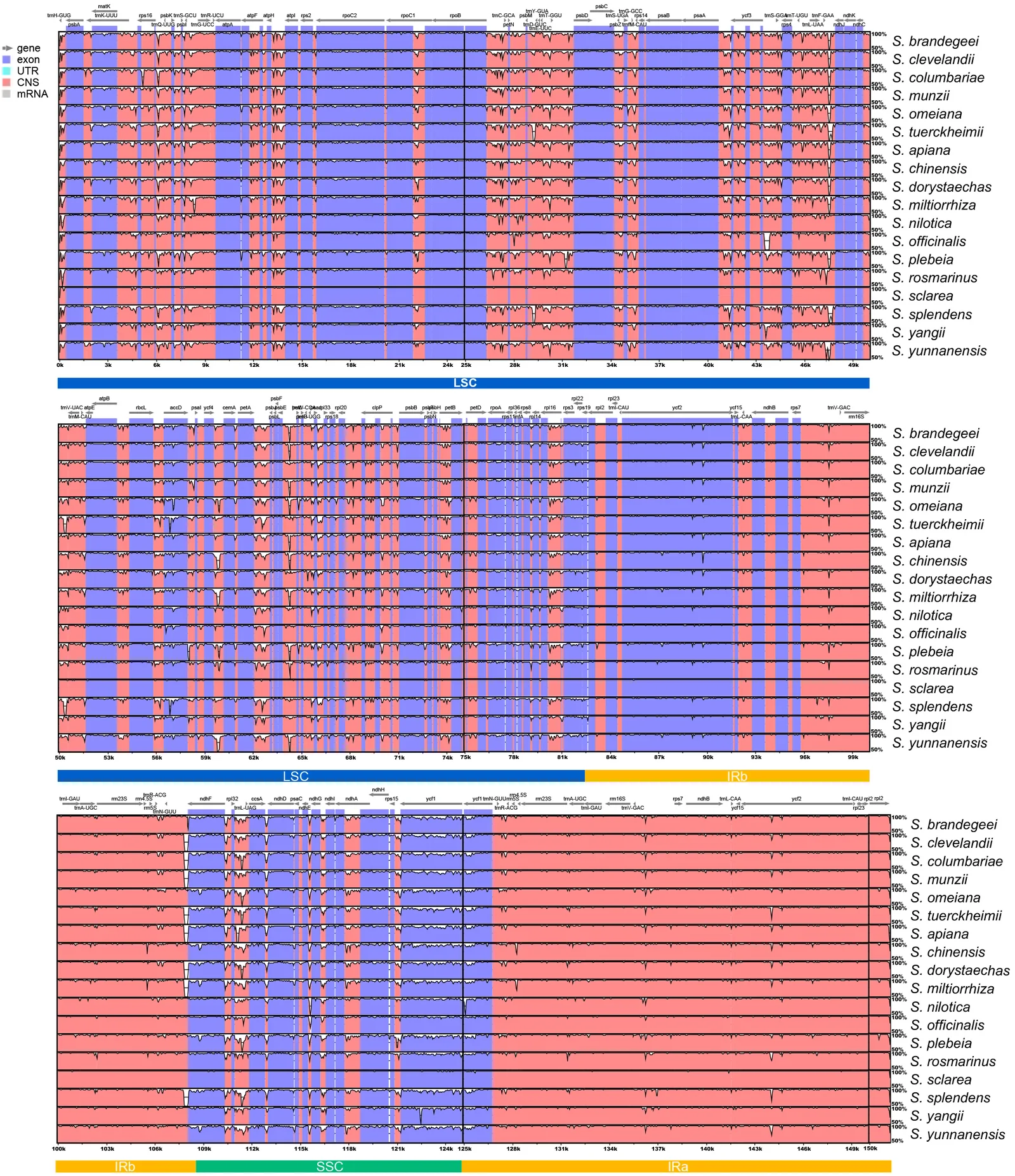
Comparative analysis of plastid genome sequences from 18 *Salvia* species using *S. aethiopis* (PV870855) as the reference. The horizontal axis represents the position along the plastid genome, and the vertical axis indicates sequence identity, ranging from 50% to 100%.

Sliding-window analysis across 98 *Salvia* plastomes recovered the same general pattern of uneven sequence divergence (**Figure 5**). High-variability windows were confined to the single-copy regions, whereas IR windows were markedly conserved. The most informative loci included the coding regions *ndhA*, *ndhF*, and *ycf1*, together with the intergenic spacers *rps16*-*trnQ-UUG*, *atpH*-*atpI*, *petN*-*psbM*, *psaA*-*ycf3*, *rpl32*-*trnL-UAG*, *ndhF*-*rpl32*, *ccsA*-*ndhD*, and *rps15*-*ycf1*. When each region was scored for its ability to separate *Salvia* species on its own, *ycf1* resolved 4,744 of 4,753 species pairs (99.81%), followed by *rpl32*-*trnL-UAG* and *matK* (4,722 pairs each, 99.35%), *petN*-*psbM* (4,720; 99.31%) and *trnH-GUG*-*psbA* (4,622 of 4,656; 99.27%), whereas *rbcL* resolved the fewest (4,639; 97.60%). Nucleotide diversity and discriminatory power were not equivalent: the most diverse region, *ccsA*-*ndhD* (pi = 0.084), is only 234 bp long and left 112 species pairs unresolved (**Supplementary Table 10**). These loci represent promising candidates for *Salvia* species discrimination and future plastid marker development.

**Figure 5:**
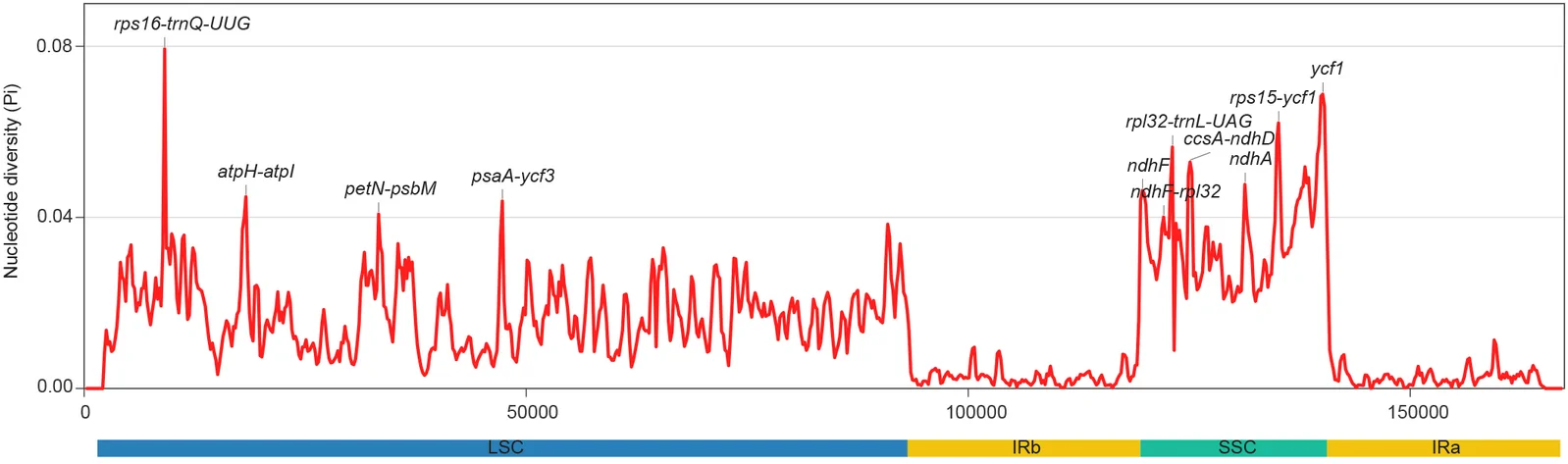
Sliding-window analysis of nucleotide diversity across the plastid genomes of 98 *Salvia* taxa. A window length of 600 bp and a step size of 200 bp were used. The x-axis shows genomic position, and the y-axis shows nucleotide diversity values for each window.

### Phylogenetic relationships inferred from plastome data

The maximum-likelihood plastome phylogeny provided a largely resolved framework for *Salvia* evolution (**Figure 6**; **Supplementary Table 7**). The dataset included 98 *Salvia* taxa spanning nine recognized subgenera, plus *M. longifolia* and *M. suaveolens* as outgroups. After removal of one IR copy and trimming of ambiguously aligned columns, the matrix comprised 120,396 aligned columns, of which 83.26% were constant and 9,406 were parsimony informative, and ModelFinder selected GTR+F+I+R4. Three major clades were recovered. Clade C1 consisted exclusively of members of subgenus *Glutinaria*. Clade C2 united species of subgenus *Calosphace* with representatives of *Audibertia* and *Dorystaechas*. Clade C3 comprised species assigned to subgenera *Salvia*, *Sclarea*, *Heterosphace*, *Rosmarinus*, and *Perovskia*. The deep topology therefore agreed well with the current broad infrageneric framework, while also clarifying relationships among several previously difficult lineages.

**Figure 6:**
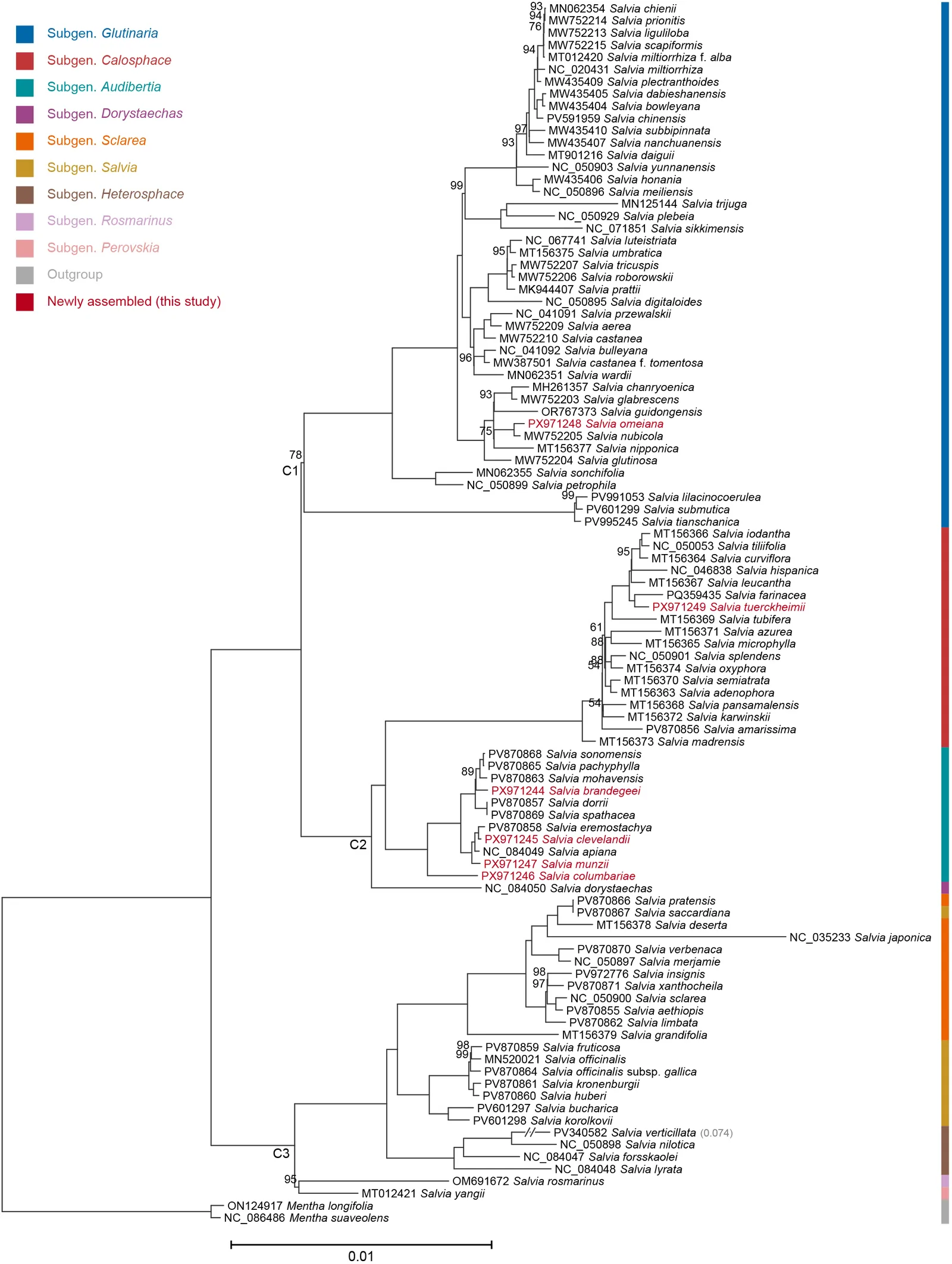
Maximum-likelihood phylogeny of 98 *Salvia* plastomes based on an IR-deduplicated, trimAl-trimmed alignment. Branch support was assessed with 1,000 ultrafast bootstrap and 1,000 SH-aLRT replicates; only ultrafast bootstrap values below 100 are shown. Red taxon names indicate the six plastomes assembled in this study, and the color bar denotes subgeneric assignment. *Mentha longifolia* and *M. suaveolens* were used as outgroups. The unusually long terminal branch of *Salvia verticillata* is shown with a break.

### Plastid RNA editing in *S. officinalis*

Analysis of the matched leaf RNA-seq and DNA-seq libraries identified 21 plastid RNA editing sites in 10 protein-coding genes of *S. officinalis* (**Figure 1**; **Table 2**; **Supplementary Table 12**). All editing events were C-to-U conversions on the coding strand of the corresponding gene, and every site was homozygous cytosine in the matched genomic library, which excludes genomic polymorphism. Most editing events were nonsynonymous (20/21, 95.2%), whereas one site was synonymous. Editing occurred most often at the second codon position (17 sites), with 3, 17 and 1 sites at the first, second and third positions respectively. Editing efficiency ranged from 24.4% to 100.0% (median 94.4%), and median read depth at the retained sites was 70x (range 21-286x). Read depth for each protein-coding gene, and the composition of the mapped plastid read depth by functional class, are given in **Supplementary Table S13** and **14**. Seven nonsynonymous sites were confirmed by Sanger sequencing of genomic DNA and cDNA amplicons prepared from leaf tissue of a separate cultivated *S. officinalis* plant: five of those listed in **Table 2**, together with the two *matK* sites discussed below. In every case the genomic sequence read C on the coding strand and the cDNA read U, or a C/U mixture at the partially edited *rpoA* - 200 site (**Figure 1b**; **Supplementary Table 8**). **Supplementary Table 12** lists all 28 candidate positions considered. Five further sites, in *atpI*, *matK* and *ndhE*, were recovered when the depth threshold was relaxed to 10 reads, and two of these, *matK* - 155 and *matK* - 1180, were confirmed here by Sanger sequencing even though the RNA-seq depth at those positions (12 and 15 reads) fell below the threshold used in the primary analysis. Two further candidates, in *ndhB* and *rps7*, met the depth requirement but carried U in only about 3% of reads; these are more likely to reflect sequencing noise than editing and are not included in the reported set. *matK* was covered at a mean depth of only 15x (**Supplementary Table 13**), in the lowest quartile of the protein-coding genes examined, which is why its sites are the ones most affected by the depth threshold.

**Table 2:** Plastid RNA editing sites identified in *Salvia officinalis*.

Gene	Strand	Plastome position	Position in gene	Codon number	Position in codon	Editing type	Codon change	Amino acid change	RNA read depth	Editing efficiency	DNA read depth	Confirmed by Sanger sequencing
*atpF*	–	12529	92	31	2	C to U	CCA to CUA	Pro to Leu	37	100.0%	1977	no
*matK*	–	2305	1249	417	1	C to U	CAU to UAU	His to Tyr	21	81.0%	1947	no
*ndhA*	–	118798	341	114	2	C to U	UCA to UUA	Ser to Leu	66	66.7%	2529	no
*ndhB*	–	94799	149	50	2	C to U	UCA to UUA	Ser to Leu	196	96.4%	5015	yes
*ndhB*	–	94481	467	156	2	C to U	CCA to CUA	Pro to Leu	197	97.0%	5073	no
*ndhB*	–	94362	586	196	1	C to U	CAU to UAU	His to Tyr	216	92.6%	4685	no
*ndhB*	–	94211	737	246	2	C to U	CCA to CUA	Pro to Leu	286	95.8%	4129	no
*ndhB*	–	94202	746	249	2	C to U	UCU to UUU	Ser to Phe	274	93.8%	4155	no
*ndhB*	–	93443	830	277	2	C to U	UCA to UUA	Ser to Leu	160	97.5%	4375	yes
*ndhB*	–	93437	836	279	2	C to U	UCA to UUA	Ser to Leu	158	86.1%	4407	yes
*ndhB*	–	92792	1481	494	2	C to U	CCA to CUA	Pro to Leu	145	97.2%	4517	no
*ndhG*	–	115740	347	116	2	C to U	CCA to CUA	Pro to Leu	23	100.0%	2606	no
*petB*	+	74186	12	4	3	C to U	GUC to GUU	Val (synonymous)	45	24.4%	2650	no
*petB*	+	74592	418	140	1	C to U	CGG to UGG	Arg to Trp	31	96.8%	3040	yes
*petB*	+	74785	611	204	2	C to U	CCA to CUA	Pro to Leu	32	96.9%	2962	no
*psbZ*	+	35074	50	17	2	C to U	UCA to UUA	Ser to Leu	70	65.7%	2443	no
*rpl23*	–	84284	71	24	2	C to U	UCU to UUU	Ser to Phe	34	97.1%	5229	no
*rpl23*	–	84266	89	30	2	C to U	UCA to UUA	Ser to Leu	41	87.8%	5122	no
*rpoA*	–	77182	200	67	2	C to U	UCC to UUC	Ser to Phe	72	66.7%	2521	yes
*rps14*	–	36190	80	27	2	C to U	UCA to UUA	Ser to Leu	66	93.9%	2281	no
*rps14*	–	36121	149	50	2	C to U	CCA to CUA	Pro to Leu	72	94.4%	2047	no

The edited genes identified in *S. officinalis* were *atpF*, *matK*, *ndhA*, *ndhB*, *ndhG*, *petB*, *psbZ*, *rpl23*, *rpoA* and *rps14*. Among these, *atpF*, *ndhA*, *ndhB*, *ndhG*, *petB* and *psbZ* are functionally associated with photosynthesis and electron transport, whereas *matK*, *rpl23*, *rpoA* and *rps14* are involved in plastid gene expression. The cytosine targeted by editing was retained at the same position in all 21 sites in at least 90% of the 98 *Salvia* plastomes (mean 99.5% of taxa, with complete conservation at 19 sites), indicating that these are conserved candidate cytosine positions across the genus rather than features peculiar to *S. officinalis*; conservation of the cytosine establishes only that the potential for editing is retained, and whether these positions are actually edited in other species cannot be determined without transcriptome data for those taxa. Together, these results suggest that RNA editing contributes to the post-transcriptional refinement of plastid gene function in *Salvia*.

## Discussion

The *Salvia* plastid genomes analyzed here were highly conserved in size, organization, GC content, and gene inventory, which is consistent with the generally stable architecture of angiosperm plastomes and with previous comparative studies in *Salvia* (Liang et al., 2019; Ruhlman and Jansen, 2021; Wu et al., 2021). The limited variation that was observed was mainly associated with minor IR/SC boundary shifts and local repeat accumulation rather than with large-scale rearrangements. This combination of structural conservatism and localized divergence explains why plastid genomes can simultaneously support deep phylogenetic inference and fine-scale marker development.

The divergence analyses also showed a familiar but informative pattern: IR regions were substantially more conserved than the single-copy regions, and non-coding spacers were generally more variable than coding regions (Wolfe et al., 1987; Li et al., 2016). The loci highlighted here, especially *ndhA*, *ndhF*, *ycf1*, *rps16*-*trnQ-UUG*, *atpH*-*atpI*, *petN*-*psbM*, *psaA*-*ycf3*, *rpl32*-*trnL-UAG*, *ccsA*-*ndhD*, *ndhF*-*rpl32*, and *rps15*-*ycf1*, largely coincide with the hotspots reported from smaller *Salvia* plastome datasets (Wu et al., 2021; Akrami et al., 2025; Du et al., 2025; Gao et al., 2026), and the fact that essentially the same regions emerge from a 98-plastome sample as from datasets of five to eighteen genomes indicates that the hotspot landscape of the genus is stable with respect to taxon sampling. Ranking the same loci by the number of species pairs they actually separate, rather than by nucleotide diversity, changes the picture: *ycf1* leaves only 9 of 4,753 *Salvia* species pairs unresolved, whereas the most diverse region, *ccsA*-*ndhD*, leaves 112 because it is only 234 bp long. Compared with traditional plastid barcodes such as *rbcL* and *matK*, which are widely used because of their universality but are relatively conserved, these hypervariable regions provide greater sequence divergence and correspondingly higher discriminatory power among closely related *Salvia* species. Accordingly, they represent promising candidate markers for species identification and germplasm authentication, although their performance in practice will also depend on primer universality and amplification success, which we did not test here.

The repeat analyses further support the practical value of plastome data for germplasm authentication. SSR abundance was moderate and showed among-species variation, and SSRs were strongly enriched in the LSC, in intergenic regions, and among A/T-rich motifs. This pattern is similar to that reported for other plant plastomes and is useful for developing polymorphic markers because intergenic A/T-rich loci often evolve faster than conserved coding regions (Provan et al., 2001; Ebert and Peakall, 2009). For a genus with extensive medicinal use and frequent problems of species substitution, the combination of SSR loci and high-divergence plastid regions provides a realistic toolbox for species identification and quality control. In practical terms, these candidate markers may be especially useful for the authentication and germplasm screening of medicinally important species such as *S. miltiorrhiza* and *S. officinalis*, in which accurate identification is important for resource utilization and product consistency. They may also support accession identification and germplasm management in economically important species such as *S. sclarea* and *S. rosmarinus*. In breeding-related contexts, plastome-derived markers may further assist maternal lineage tracing when used together with other genomic data.

The phylogenomic analysis based on 98 plastomes reinforces the value of plastome-scale data for resolving major *Salvia* lineages. The same three clades were recovered whether the analysis used complete plastomes, IR-deduplicated and trimmed alignments, or a partitioned supermatrix of 50 protein-coding genes (**Supplementary Figure S4** and S5; Robinson-Foulds distances of 12 to 24 among the three topologies, 87.6% to 93.8% of bipartitions shared), which indicates that the backbone is not an artefact of any single matrix construction. Support for clade C1 was nevertheless sensitive to these choices, falling from SH-aLRT 96.2/bootstrap 97 in the untrimmed complete-plastome matrix to 55.0/78 after inverted-repeat deduplication and trimming, so we regard the monophyly of subgenus *Glutinaria* as recovered but not strongly supported by plastid data alone. The recovered topology broadly supports the current subgeneric framework proposed for *Salvia* sensu lato (Drew et al., 2017; Hu et al., 2018; Kriebel et al., 2019). In particular, the clear separation of *Glutinaria* and the close association among *Calosphace*, *Audibertia*, and *Dorystaechas* are congruent with previous phylogenetic work based on more limited datasets (Jenks et al., 2013; Will and Classen-Bockhoff, 2017). The three clades also correspond to broad geographic groupings. Clade C1, comprising subgenus *Glutinaria*, is almost entirely East Asian. Clade C2 unites the New World subgenera *Calosphace* and *Audibertia* with the eastern Mediterranean *Dorystaechas*. Clade C3 assembles the Old World subgenera *Salvia*, *Sclarea* and *Heterosphace* together with *Rosmarinus* and *Perovskia*, and is centered on the Mediterranean and Irano-Turanian regions. This correspondence between plastid lineage and geographic area is consistent with earlier biogeographic analyses of the genus (Kriebel et al., 2019; Rose et al., 2021), although the present data do not include divergence-time estimation or ancestral-area reconstruction and therefore cannot be used to infer dispersal histories. Although plastid genomes alone cannot capture the full complexity of hybridization or incomplete lineage sorting, the present topology provides a plastome-based framework for taxonomy, conservation planning, and comparative trait studies in the genus.

The RNA editing profile of *S. officinalis* adds a post-transcriptional dimension to plastid genome evolution in *Salvia*. A total of 21 editing sites were identified in 10 protein-coding genes, of which 20 were nonsynonymous, and seven of the nonsynonymous sites were confirmed by Sanger sequencing of both gDNA and cDNA. Most edits were C-to-U conversions at the second codon position and most were nonsynonymous, consistent with the general features of plastid RNA editing in land plants (Small et al., 2020; Duan et al., 2023). Across the 98 *Salvia* plastomes the edited cytosines were themselves highly conserved (mean 99.5% of taxa retaining the C at the same position, with complete conservation at 19 of 21 sites), so the positions recovered here are ones at which the genus as a whole retains the potential for C-to-U editing; whether they are actually edited in other species cannot be established without transcriptome data for those taxa. The edited genes identified here are mainly involved in plastid gene expression and photosynthetic electron transport, suggesting that RNA editing may contribute to the post-transcriptional recoding of plastid transcripts in *S. officinalis*. Although such effects may indirectly influence the metabolic background of secondary metabolite accumulation, the present data do not permit a direct functional link to specific biosynthetic pathways in *Salvia*. In addition, the detection of synonymous edits indicates that some editing events may have roles beyond amino acid replacement, potentially affecting RNA stability or translation efficiency (Lo Giudice et al., 2019). The number of plastid editing sites recovered here is far lower than the approximately 451 sites reported for the *S. officinalis* mitochondrial transcriptome (Yang et al., 2023), but this contrast is expected rather than anomalous: in land plants the mitochondrial genome typically carries an order of magnitude more editing sites than the plastid genome, and the two compartments are not directly comparable. The low per-site depths reported previously were partly a consequence of this library composition and partly an artefact of the earlier analysis, which did not pool the two inverted-repeat copies of *ndhB* and reported the depth of a generic variant call rather than the depth available at the site. With the inverted repeat pooled and no depth cap applied, median depth at the retained sites is 70x. Per-gene depth, rather than library size, sets the limit on how many sites can be detected: mean coverage across protein-coding genes ranged from 1.3x in *rpoC2* to 9,832x in *psaJ*, and *matK*, which carries two of the Sanger-confirmed sites, lies in the lowest quartile at 15x. Relaxing the depth threshold from 20 to 10 reads recovered five further candidates, two of which are confirmed by our own Sanger sequencing, so the set reported here should be read as a conservative lower bound rather than a complete inventory.

This study also has several limitations that should be acknowledged. No new plant material was collected for the plastome assemblies; all the high-throughput sequencing data analyzed here are public, and species identities rest on the determinations made by the original collectors, supported here by the molecular comparison of *S. columbariae* with an independently published conspecific plastome. The only newly collected material is the leaf tissue of the single cultivated *S. officinalis* plant used for Sanger validation, which is a different individual from the one that generated the public RNA-seq and DNA-seq libraries; the Sanger data therefore confirm that these positions are edited in the species but cannot be used to verify the editing frequencies measured from SRR5150710. Although the 18 plastid genomes compared in detail represent major *Salvia* lineages, they cover only part of the genus, and the whole-genome visual comparisons were restricted to this subset for legibility rather than for lack of data. In addition, plastid RNA editing was characterized only in *S. officinalis*. This species was selected because matched DNA and RNA datasets from the same BioSample were available and the nonsynonymous sites could be validated by Sanger sequencing; however, the resulting dataset does not yet allow broader evaluation of the evolutionary conservation or species specificity of plastid RNA editing across *Salvia*, and the RNA library was total RNA rather than ribosomal-RNA-depleted, so lowly expressed coding regions are represented less well than highly expressed ones. Finally, because plastid genomes are typically maternally inherited, plastome-based phylogenies primarily reflect the maternal lineage and may not fully represent species history where hybridization, introgression, or incomplete lineage sorting has occurred. Future studies combining denser taxon sampling, ribosomal-RNA-depleted transcriptomes, multi-species RNA editing comparisons, and nuclear genomic data will help provide a more complete view of *Salvia* evolution and molecular diversity.

## Conclusion

This study provides a comprehensive plastid genomic framework for *Salvia*. The *Salvia* plastomes compared here were structurally conservative but contained repeat variation and divergence hotspots suitable for marker development, and the discriminatory power of the leading candidate regions was quantified against the standard plastid barcodes. Expanded phylogenomic sampling, analyzed after removing inverted-repeat redundancy and trimming ambiguously aligned columns, resolved three major *Salvia* clades and strengthened the plastome-based backbone for infrageneric classification. In parallel, plastid RNA editing analysis in *S. officinalis* revealed a set of 21 editing events dominated by nonsynonymous C-to-U conversions and corresponding closely to the conserved editing positions known from other angiosperms. These results provide useful genomic resources for species authentication, phylogenetic reconstruction, germplasm management, and the future exploitation of medicinal and horticultural *Salvia* species.

## Supporting information

Supplementary data are provided with this manuscript.

## Data Availability

The datasets presented in this study can be found in online repositories. The names of the repository/repositories and accession number(s) can be found in the article/**Supplementary Material**.

## Supplementary material

Supplementary Figure 1 Circular maps of the plastid genomes of the five remaining *Salvia* species assembled in this study: **(a)** *S. brandegeei* (PX971244; SRR29086339), **(b)** *S. clevelandii* (PX971245; SRR29086354), **(c)** *S. munzii* (PX971247; SRR29086340), **(d)** *S. omeiana* (PX971248; SRR16693773) and **(e)** *S. tuerckheimii* (PX971249; SRR16693719). Genes on the inside of each circle are transcribed clockwise and those on the outside counterclockwise; colors denote functional groups.

Supplementary Figure 2 Characterization of repetitive elements in the plastid genomes of *Salvia* species. **(a)** Number of SSRs classified by repeat motif type and copy number; the six pure repeat classes are plotted and the 23 compound loci listed in **Supplementary Tables 4** and **5** are not shown. **(b)** Relative distribution of SSRs among the LSC, SSC, and IR regions. **(c)** Composition and abundance of long-repeat types. **(d)** Size distribution of long repeats.

Supplementary Figure 3 Comparison of the junctions among large single copy (LSC), small single copy (SSC), and inverted repeat (IR) regions in the plastid genomes of *Salvia* species.

Supplementary Figure 4 Maximum-likelihood phylogeny of 98 *Salvia* plastomes inferred from complete plastome sequences with both inverted-repeat copies retained and without alignment trimming. Ultrafast bootstrap values are shown only for nodes with support below 100%. Taxa shown in red indicate plastomes assembled in this study. The terminal branch of *Salvia verticillata* is shown with a break because of its greater length.

Supplementary Figure 5 Maximum-likelihood phylogeny of 98 *Salvia* plastomes inferred from a partitioned supermatrix of 50 protein-coding genes present in single copy across all 100 taxa. Ultrafast bootstrap values are shown only for nodes with support below 100%. Taxa shown in red indicate plastomes assembled in this study.
