# Supplementary material for: Comparative plastid genomics reveals phylogenetic relationships and candidate molecular markers in Salvia
Source: Front Plant Sci. 2026 Sep 14;17:1932456. doi: 10.3389/fpls.2026.1932456 (PMC13618359; doi:10.3389/fpls.2026.1932456)
Supplement: Supplementary file 1 [file DataSheet1.pdf]

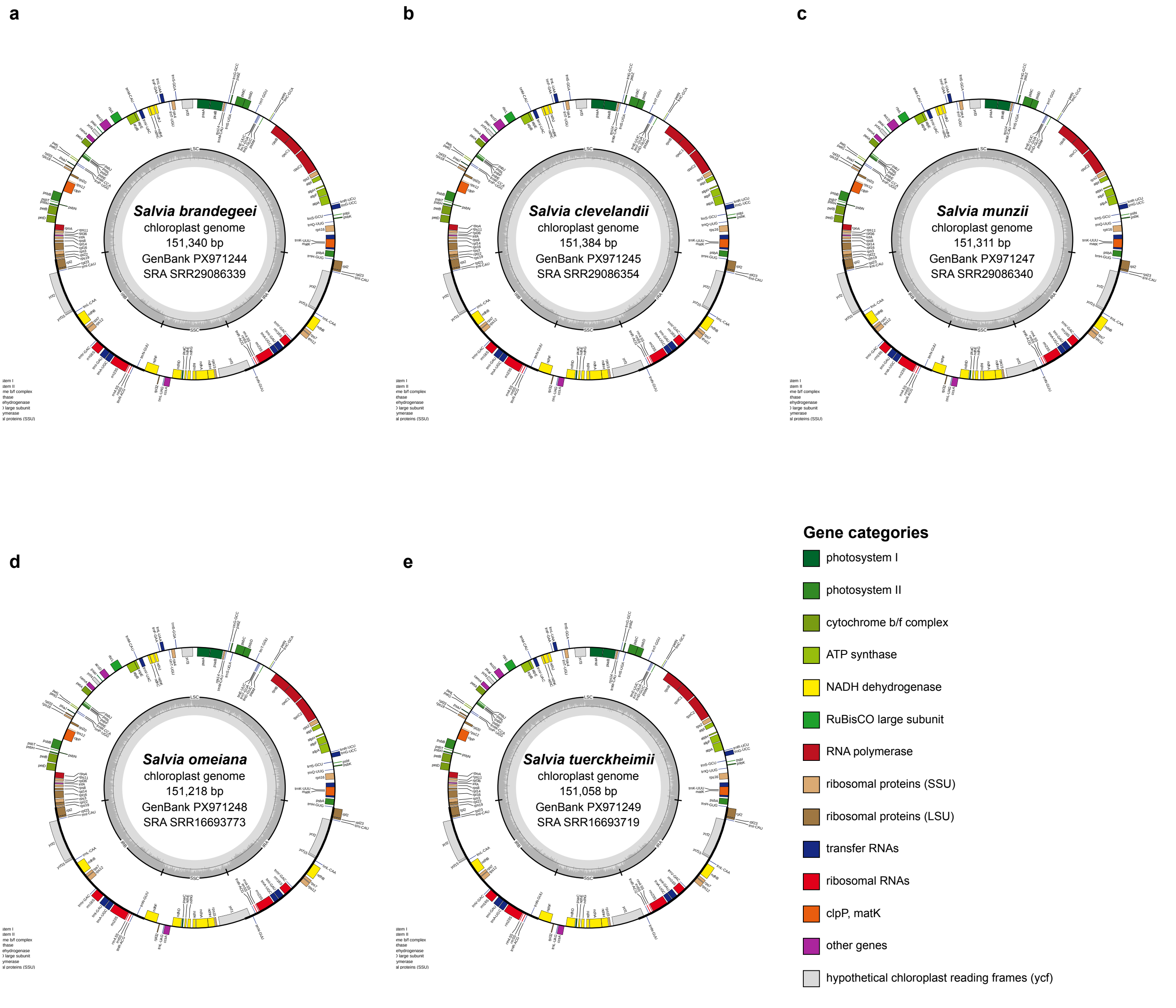
a
Salvia brandegeei
chloroplast genome
151,340 bp
GenBank PX971244
SRA SRR29086339
b
Salvia clevelandii
chloroplast genome
151,384 bp
GenBank PX971245
SRA SRR29086354
c
Salvia munzii
chloroplast genome
151,311 bp
GenBank PX971247
SRA SRR29086340
d
Salvia omeiana
chloroplast genome
151,218 bp
GenBank PX971248
SRA SRR16693773
e
Salvia tuerckheimii
chloroplast genome
151,058 bp
GenBank PX971249
SRA SRR16693719
Gene categories
photosystem I
photosystem II
cytochrome b/f complex
ATP synthase
NADH dehydrogenase
RuBisCO large subunit
RNA polymerase
ribosomal proteins (SSU)
ribosomal proteins (LSU)
transfer RNAs
ribosomal RNAs
clpP, matK
other genes
hypothetical chloroplast reading frames (ycf)
