# Supplementary material for: Comparative plastid genomics reveals phylogenetic relationships and candidate molecular markers in Salvia
Source: Front Plant Sci. 2026 Sep 14;17:1932456. doi: 10.3389/fpls.2026.1932456 (PMC13618359; doi:10.3389/fpls.2026.1932456)
Supplement: Supplementary file 2 [file DataSheet2.pdf]

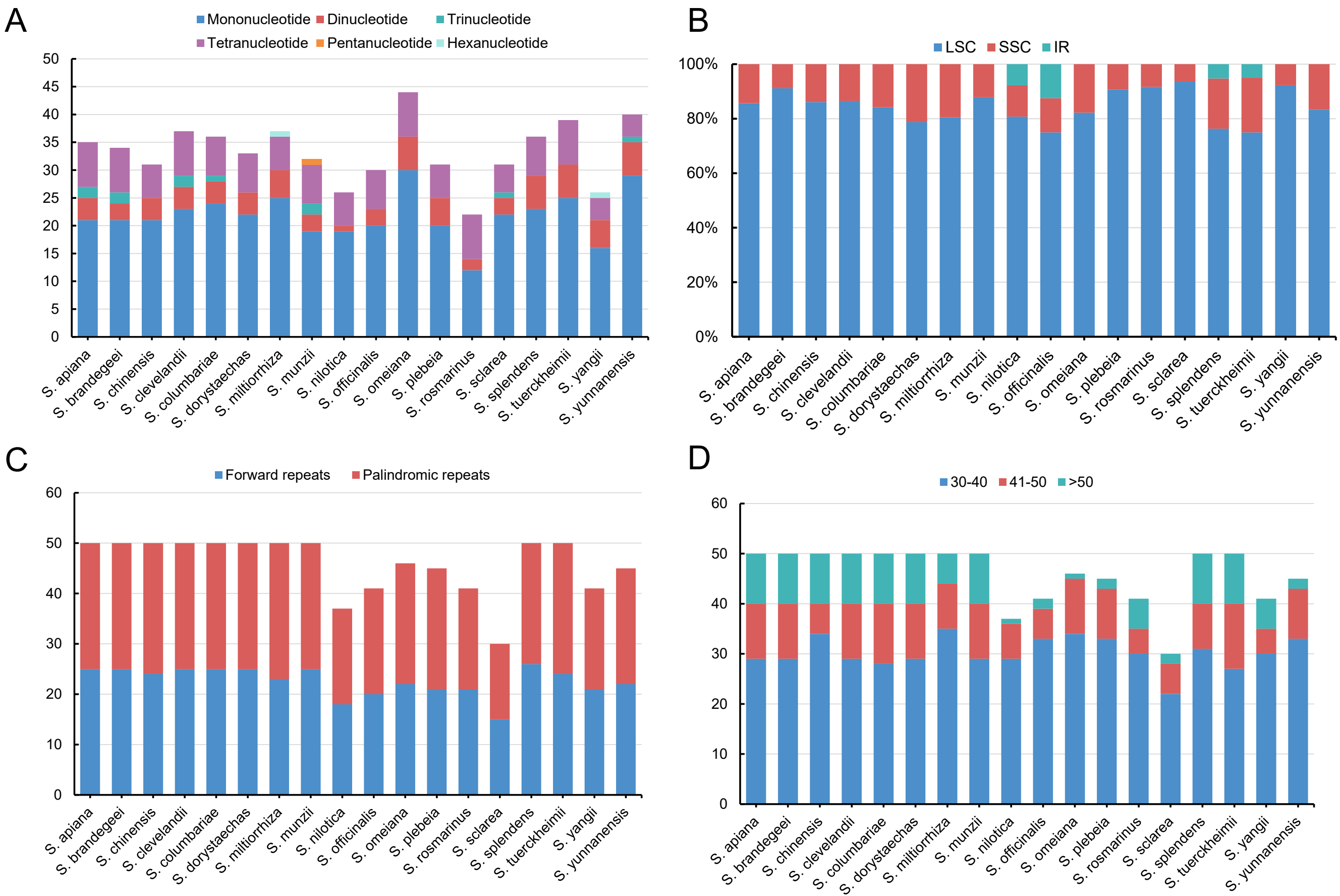
A
Mononucleotide
Dinucleotide
Trinucleotide
Tetranucleotide
Pentanucleotide
Hexanucleotide
B
LSC
SSC
IR
C
Forward repeats
Palindromic repeats
D
30-40
41-50
>50
S. apiana
S. brandegeei
S. chinensis
S. clevelandii
S. columbariae
S. dorystaechas
S. miltiorrhiza
S. munzii
S. nilotica
S. officinalis
S. omeiana
S. plebeia
S. rosmarinus
S. sclarea
S. splendens
S. tuerckheimii
S. yangii
S. yunnanensis
