# Supplementary material for: Comparative plastid genomics reveals phylogenetic relationships and candidate molecular markers in Salvia
Source: Front Plant Sci. 2026 Sep 14;17:1932456. doi: 10.3389/fpls.2026.1932456 (PMC13618359; doi:10.3389/fpls.2026.1932456)
Supplement: Supplementary file 3 [file DataSheet3.pdf]

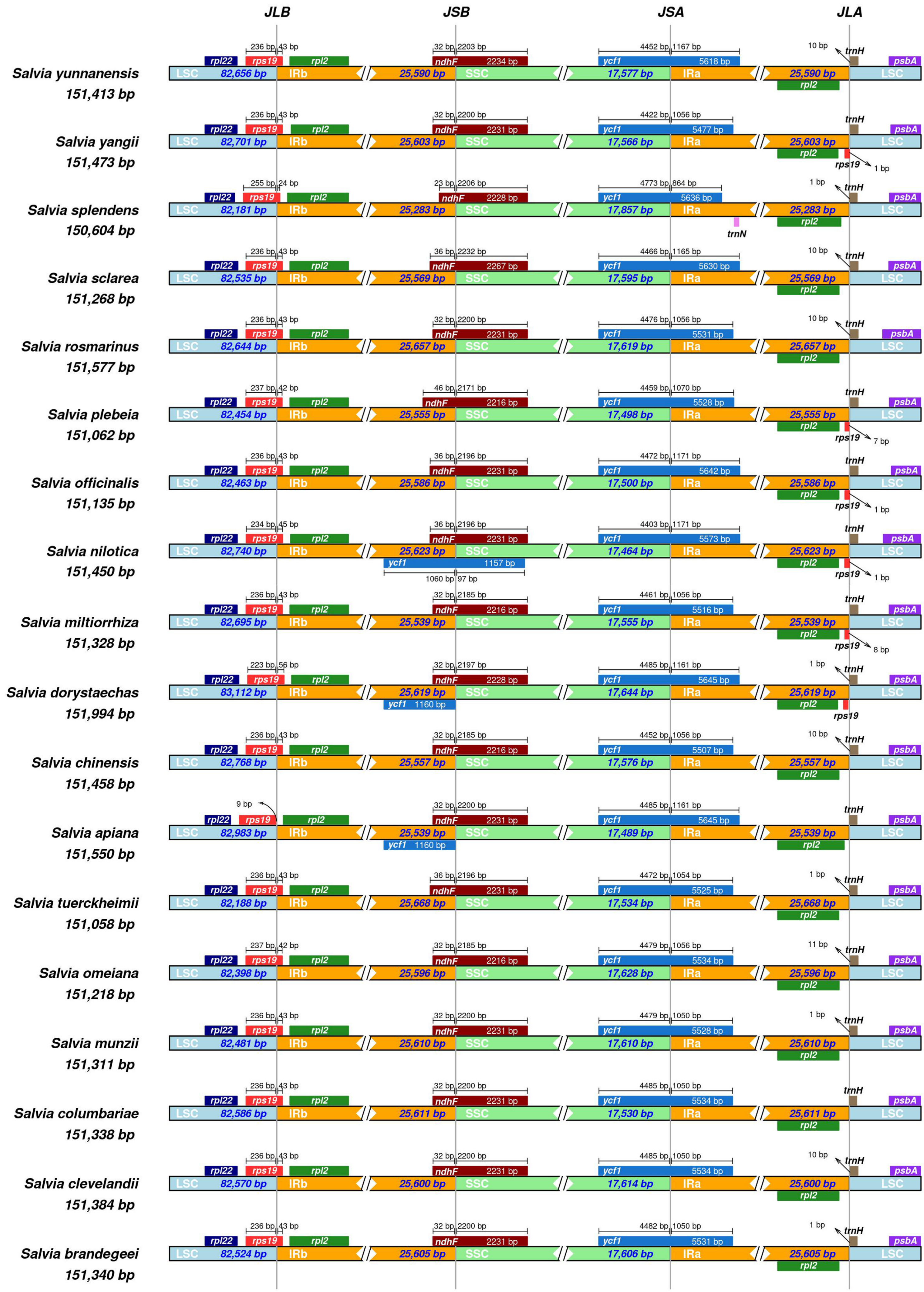
JLB
JSB
JSA
JLA
Salvia yunnanensis 151,413 bp
236 bp 43 bp rpl22 rps19 rpl2 LSC 82,656 bp IRb
32 bp 2203 bp ndhF 2234 bp 25,590 bp SSC
4452 bp 1167 bp ycf1 5618 bp 17,577 bp IRa
10 bp trnH psbA 25,590 bp LSC rpl2
Salvia yangii 151,473 bp
236 bp 43 bp rpl22 rps19 rpl2 LSC 82,701 bp IRb
32 bp 2200 bp ndhF 2231 bp 25,603 bp SSC
4422 bp 1056 bp ycf1 5477 bp 17,566 bp IRa
trnH psbA 25,603 bp LSC rpl2 rps19 1 bp
Salvia splendens 150,604 bp
255 bp 24 bp rpl22 rps19 rpl2 LSC 82,181 bp IRb
23 bp 2206 bp ndhF 2228 bp 25,283 bp SSC
4773 bp 864 bp ycf1 5636 bp 17,857 bp IRa trnN
1 bp trnH psbA 25,283 bp LSC rpl2
Salvia sclarea 151,268 bp
236 bp 43 bp rpl22 rps19 rpl2 LSC 82,535 bp IRb
36 bp 2232 bp ndhF 2267 bp 25,569 bp SSC
4466 bp 1165 bp ycf1 5630 bp 17,595 bp IRa
10 bp trnH psbA 25,569 bp LSC rpl2
Salvia rosmarinus 151,577 bp
236 bp 43 bp rpl22 rps19 rpl2 LSC 82,644 bp IRb
32 bp 2200 bp ndhF 2231 bp 25,657 bp SSC
4476 bp 1056 bp ycf1 5531 bp 17,619 bp IRa
10 bp trnH psbA 25,657 bp LSC rpl2
Salvia plebeia 151,062 bp
237 bp 42 bp rpl22 rps19 rpl2 LSC 82,454 bp IRb
46 bp 2171 bp ndhF 2216 bp 25,555 bp SSC
4459 bp 1070 bp ycf1 5528 bp 17,498 bp IRa
trnH psbA 25,555 bp LSC rpl2 rps19 7 bp
Salvia officinalis 151,135 bp
236 bp 43 bp rpl22 rps19 rpl2 LSC 82,463 bp IRb
36 bp 2196 bp ndhF 2231 bp 25,586 bp SSC
4472 bp 1171 bp ycf1 5642 bp 17,500 bp IRa
trnH psbA 25,586 bp LSC rpl2 rps19 1 bp
Salvia nilotica 151,450 bp
234 bp 45 bp rpl22 rps19 rpl2 LSC 82,740 bp IRb
36 bp 2196 bp ndhF 2231 bp 25,623 bp SSC ycf1 1157 bp 1060 bp 97 bp
4403 bp 1171 bp ycf1 5573 bp 17,464 bp IRa
trnH psbA 25,623 bp LSC rpl2 rps19 1 bp
Salvia miltiorrhiza 151,328 bp
236 bp 43 bp rpl22 rps19 rpl2 LSC 82,695 bp IRb
32 bp 2185 bp ndhF 2216 bp 25,539 bp SSC
4461 bp 1056 bp ycf1 5516 bp 17,555 bp IRa
trnH psbA 25,539 bp LSC rpl2 rps19 8 bp
Salvia dorystaechas 151,994 bp
223 bp 56 bp rpl22 rps19 rpl2 LSC 83,112 bp IRb
32 bp 2197 bp ndhF 2228 bp 25,619 bp SSC ycf1 1160 bp
4485 bp 1161 bp ycf1 5645 bp 17,644 bp IRa
1 bp trnH psbA 25,619 bp LSC rpl2 rps19
Salvia chinensis 151,458 bp
236 bp 43 bp rpl22 rps19 rpl2 LSC 82,768 bp IRb
32 bp 2185 bp ndhF 2216 bp 25,557 bp SSC
4452 bp 1056 bp ycf1 5507 bp 17,576 bp IRa
10 bp trnH psbA 25,557 bp LSC rpl2
Salvia apiana 151,550 bp
9 bp rpl22 rps19 rpl2 LSC 82,983 bp IRb
32 bp 2200 bp ndhF 2231 bp 25,539 bp SSC ycf1 1160 bp
4485 bp 1161 bp ycf1 5645 bp 17,489 bp IRa
trnH psbA 25,539 bp LSC rpl2
Salvia tuerckheimii 151,058 bp
236 bp 43 bp rpl22 rps19 rpl2 LSC 82,188 bp IRb
36 bp 2196 bp ndhF 2231 bp 25,668 bp SSC
4472 bp 1054 bp ycf1 5525 bp 17,534 bp IRa
1 bp trnH psbA 25,668 bp LSC rpl2
Salvia omeiana 151,218 bp
237 bp 42 bp rpl22 rps19 rpl2 LSC 82,398 bp IRb
32 bp 2185 bp ndhF 2216 bp 25,596 bp SSC
4479 bp 1056 bp ycf1 5534 bp 17,628 bp IRa
11 bp trnH psbA 25,596 bp LSC rpl2
Salvia munzii 151,311 bp
236 bp 43 bp rpl22 rps19 rpl2 LSC 82,481 bp IRb
32 bp 2200 bp ndhF 2231 bp 25,610 bp SSC
4479 bp 1050 bp ycf1 5528 bp 17,610 bp IRa
1 bp trnH psbA 25,610 bp LSC rpl2
Salvia columbariae 151,338 bp
236 bp 43 bp rpl22 rps19 rpl2 LSC 82,586 bp IRb
32 bp 2200 bp ndhF 2231 bp 25,611 bp SSC
4485 bp 1050 bp ycf1 5534 bp 17,530 bp IRa
trnH psbA 25,611 bp LSC rpl2
Salvia clevelandii 151,384 bp
236 bp 43 bp rpl22 rps19 rpl2 LSC 82,570 bp IRb
32 bp 2200 bp ndhF 2231 bp 25,600 bp SSC
4485 bp 1050 bp ycf1 5534 bp 17,614 bp IRa
10 bp trnH psbA 25,600 bp LSC rpl2
Salvia brandegeei 151,340 bp
236 bp 43 bp rpl22 rps19 rpl2 LSC 82,524 bp IRb
32 bp 2200 bp ndhF 2231 bp 25,605 bp SSC
4482 bp 1050 bp ycf1 5531 bp 17,606 bp IRa
1 bp trnH psbA 25,605 bp LSC rpl2
