# Supplementary material for: Comparative plastid genomics reveals phylogenetic relationships and candidate molecular markers in Salvia
Source: Front Plant Sci. 2026 Sep 14;17:1932456. doi: 10.3389/fpls.2026.1932456 (PMC13618359; doi:10.3389/fpls.2026.1932456)
Supplement: Supplementary file 5 [file DataSheet5.pdf]

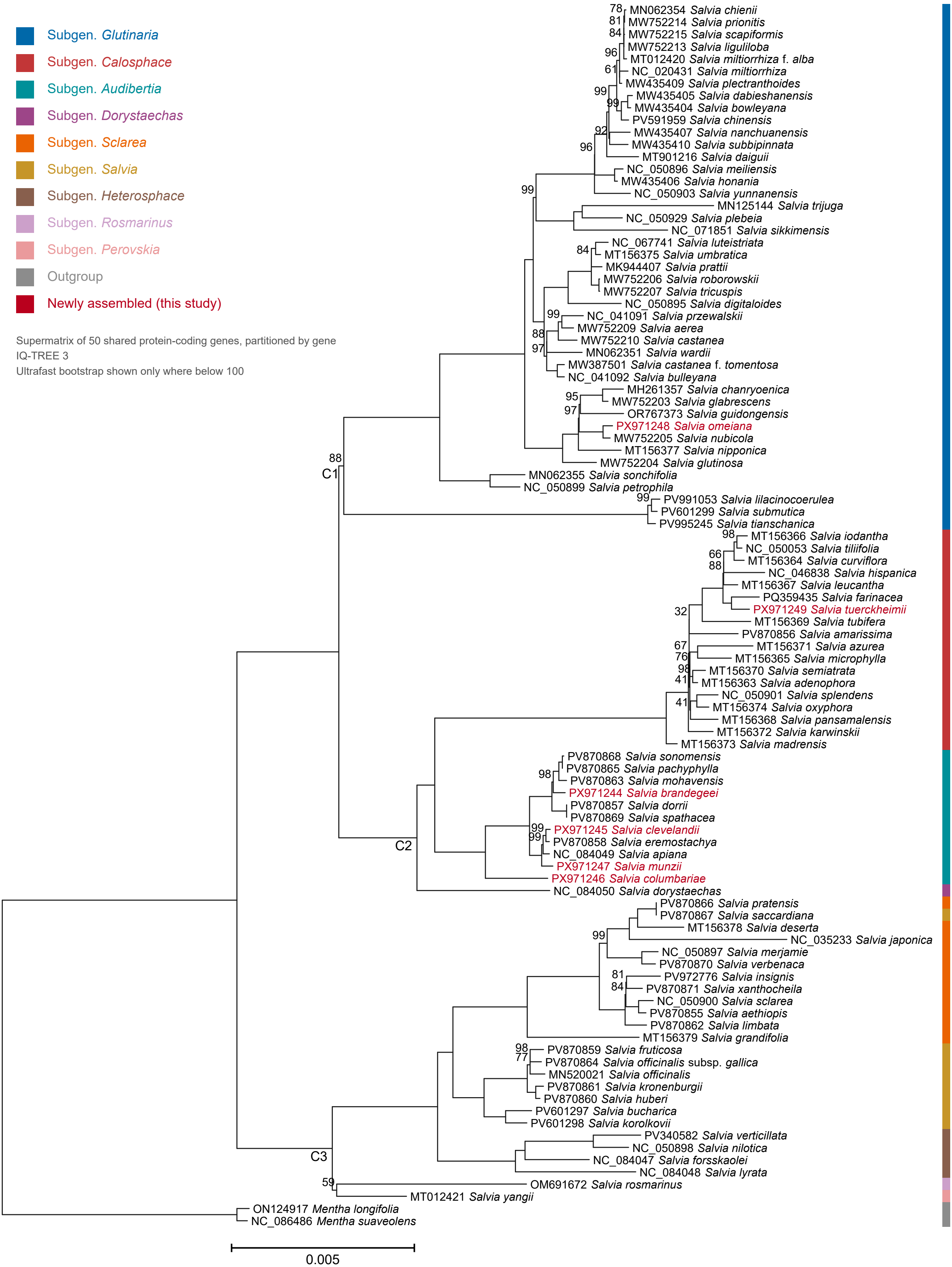
Subgen. Glutinaria
Subgen. Calosphace
Subgen. Audibertia
Subgen. Dorystaechas
Subgen. Sclarea
Subgen. Salvia
Subgen. Heterosphace
Subgen. Rosmarinus
Subgen. Perovskia
Outgroup
Newly assembled (this study)
Supermatrix of 50 shared protein-coding genes, partitioned by gene
IQ-TREE 3
Ultrafast bootstrap shown only where below 100
MN062354 Salvia chienii
MW752214 Salvia prionitis
MW752215 Salvia scapiformis
MW752213 Salvia liguliloba
MT012420 Salvia miltiorrhiza f. alba
NC_020431 Salvia miltiorrhiza
MW435409 Salvia plectranthoides
MW435405 Salvia dabieshanensis
MW435404 Salvia bowleyana
PV591959 Salvia chinensis
MW435407 Salvia nanchuanensis
MW435410 Salvia subbipinnata
MT901216 Salvia daiguii
NC_050896 Salvia meiliensis
MW435406 Salvia honania
NC_050903 Salvia yunnanensis
MN125144 Salvia trijuga
NC_050929 Salvia plebeia
NC_071851 Salvia sikkimensis
NC_067741 Salvia luteistriata
MT156375 Salvia umbratica
MK944407 Salvia prattii
MW752206 Salvia roborowskii
MW752207 Salvia tricuspis
NC_050895 Salvia digitaloides
NC_041091 Salvia przewalskii
MW752209 Salvia aerea
MW752210 Salvia castanea
MN062351 Salvia wardii
MW387501 Salvia castanea f. tomentosa
NC_041092 Salvia bulleyana
MH261357 Salvia chanryoenica
MW752203 Salvia glabrescens
OR767373 Salvia guidongensis
PX971248 Salvia omeiana
MW752205 Salvia nubicola
MT156377 Salvia nipponica
MW752204 Salvia glutinosa
C1
MN062355 Salvia sonchifolia
NC_050899 Salvia petrophila
PV991053 Salvia lilacinocoerulea
PV601299 Salvia submutica
PV995245 Salvia tianschanica
MT156366 Salvia iodantha
NC_050053 Salvia tiliifolia
MT156364 Salvia curviflora
NC_046838 Salvia hispanica
MT156367 Salvia leucantha
PQ359435 Salvia farinacea
PX971249 Salvia tuerckheimii
MT156369 Salvia tubifera
PV870856 Salvia amarissima
MT156371 Salvia azurea
MT156365 Salvia microphylla
MT156370 Salvia semiatrata
MT156363 Salvia adenophora
NC_050901 Salvia splendens
MT156374 Salvia oxyphora
MT156368 Salvia pansamalensis
MT156372 Salvia karwinskii
MT156373 Salvia madrensis
PV870868 Salvia sonomensis
PV870865 Salvia pachyphylla
PV870863 Salvia mohavensis
PX971244 Salvia brandegeei
PV870857 Salvia dorrii
PV870869 Salvia spathacea
PX971245 Salvia clevelandii
PV870858 Salvia eremostachya
NC_084049 Salvia apiana
PX971247 Salvia munzii
C2
PX971246 Salvia columbariae
NC_084050 Salvia dorystaechas
PV870866 Salvia pratensis
PV870867 Salvia saccardiana
MT156378 Salvia deserta
NC_035233 Salvia japonica
NC_050897 Salvia merjamie
PV870870 Salvia verbenaca
PV972776 Salvia insignis
PV870871 Salvia xanthocheila
NC_050900 Salvia sclarea
PV870855 Salvia aethiopis
PV870862 Salvia limbata
MT156379 Salvia grandifolia
PV870859 Salvia fruticosa
PV870864 Salvia officinalis subsp. gallica
MN520021 Salvia officinalis
PV870861 Salvia kronenburgii
PV870860 Salvia huberi
PV601297 Salvia bucharica
PV601298 Salvia korolkovii
PV340582 Salvia verticillata
NC_050898 Salvia nilotica
NC_084047 Salvia forsskaolei
NC_084048 Salvia lyrata
C3
OM691672 Salvia rosmarinus
MT012421 Salvia yangii
ON124917 Mentha longifolia
NC_086486 Mentha suaveolens
0.005
